# *Helicobacter pylori* infection, gestational diabetes mellitus and insulin resistance among pregnant Sudanese women

**DOI:** 10.1186/s13104-018-3642-9

**Published:** 2018-07-28

**Authors:** Shimos A. Alshareef, Duria A. Rayis, Ishag Adam, Gasim I. Gasim

**Affiliations:** 1 Faculty of Medicine, University of Khartoum, Khartoum, Sudan; 2 Faculty of Medicine, Alneelain University, Khartoum, Sudan

**Keywords:** Insulin resistance, Pregnancy, *H. pylori*, *Sudan*

## Abstract

**Objectives:**

To assess the association between *Helicobacter pylori* (*H. pylori*) infection and insulin resistance among pregnant Sudanese women attending Saad Abuelela hospital (Khartoum). A cross-sectional study was conducted from 1st July 2017 to 31st January 2018. One hundred and sixty-six women were enrolled and underwent testing for *H. pylori* IgG antibodies using specific ELISA kits. The Quantitative insulin sensitivity check index (QUICKI) was computed from the fasting insulin and glucose levels.

**Results:**

Median age, gravidity and gestational age were 27 years, 2 and 26 weeks, respectively. Twenty (12%) women were found to have gestational diabetes mellitus (GDM). *H. pylori* IgG seroprevalence was 66.0% among the study population. Univariate analysis showed that *H. pylori*-seropositivity was significantly higher among women who have GDM while Log (Homeostatic Model Assessment-β) HOMA-B% was lower (P value = 0.038, and 0.028) respectively. There was no difference between the GDM group and the other group in terms of demographics, body mass index, haemoglobin and QUICKI index results. In multivariate analysis, a higher prevalence of *H. pylori* was associated with GDM (OR = 2.8, 95% CI  1.1–7.5, P = 0.036). The current study concludes that an increased prevalence of *H. pylori* is a risk factor for the development of GDM.

## Introduction

The gram-negative bacillus *Helicobacter pylori* (*H. pylori*) is the main cause of chronic gastric infection [1]. It is estimated that fifty percent of the world population is infected with *H. pylori*, with a higher prevalence in the developing countries [2, 3]. It is not only regarded as pathogenic but also a high class carcinogen [4]. While the majority of patients infected with *H. pylori* tend to develop chronic gastritis, some of them progress to gastric and duodenal ulcers, atrophic gastritis, mucosa-associated lymphoid tissue lymphoma, intestinal metaplasia and gastric cancer [5–7].

A number of reports have observed the association between *H. pylori* and cardiovascular disease and the metabolic syndrome [8, 9]. Furthermore, *H. pylori* infection has also been indirectly linked to toll-like receptors activation via lipopolysaccharides, leading to metabolic changes culminating in insulin resistance [10]. The association between *H. pylori* infection and insulin resistance remains controversial. While some studies reported the association between *H. pylori* infection and insulin resistance [11–16], others refuted such a relationship [17–19]. Cardaropoli et al. described a strong link between gestational diabetes and *H. pylori* [20]. Furthermore, few of these studies investigated the association between *H. pylori* and insulin resistance among pregnant women. Therefore, further research addressing the interplay between *H. pylori* and insulin resistance is needed.

In Sudan, there is high prevalence of *H. pylori* among pregnant women regardless of their age and parity [21, 22]. The current study aimed to investigate the association between *H. pylori* infection and insulin resistance among pregnant Sudanese women.

## Main text

A cross-sectional study was conducted at Saad Abuelela hospital, Khartoum, Sudan from the 1st of July 2017 to 31st of January 2018. After signing an informed consent, women with single viable pregnancies were included in the study. Women with twins, diabetes, hypertension, lipid disorders, kidney disease and hemolytic anemia/thalassemia/haemoglobin variants were excluded. Data was collected using a questionnaire that included demographic, obstetrics and clinical data (age, residence, education, occupation, gravidity, parity). The women’s weight and height were measured using standard methods and then their body mass indices were computed.

The glucose tolerance test was performed at 24–28 weeks of gestation. The diagnosis of gestational diabetes mellitus was based on the recommendations of International Association of Diabetes and Pregnancy Study Groups (IADPSG), “fasting blood glucose (FBG) ≥ 92 mg/dl or 1-h blood glucose was ≥ 180 mg/dl and/or 2-h blood glucose ≥ 153 mg/dl, after a 75-g oral glucose load [23].

The glucose level was measured by the glucose oxidase method (Shino-Test Corp. Tokyo, Japan).

The fasting insulin was measured using immunoassay analyzer (AIA 360, Tosoh, Tokyo, Japan. Insulin resistance was calculated using the quantitative insulin sensitivity check index (QUICKI) = 1/[log fasting insulin level (µU/ml) + log fasting glucose level (mg/dl)] [24]. Insulin secretion was indicated by the HOMA of β-cell function (HOMA-β) (%) = 360 × fasting insulin level (µU/ml)/[fasting glucose level (mg/dl) − 63] [25].

*Helicobacter pylori* IgG antibodies were tested using commercial *H. pylori*-specific ELISA (Euroimmun, Lu¨beck, Germany). The tests were performed as per the manufacturer’s instructions. Five millilitres of blood were withdrawn in a plain tube, allowed to clot, centrifuged and stored at − 20 °C. Test values of ≥ 1.1 units were regarded as positive. Those ranging between 0.9 and 1.1 units were regarded as weakly positive and those ≤ 0.9 units were considered as negative for *H*. *pylori.*

### Statistics

SPSS for Windows (version 20.0) was used for data analyses. After checking for normality of distribution of data, the studied variables were described as means, medians and percentages. The difference of medians and proportions were compared between the *H. pylori* IgG seropositive and IgG seronegative using Mann–Whitney U test and χ^2^, respectively. All variables were entered into a logistic regression model where the dependent variable was gestational diabetes presence or absence. A P value less than 0.05 was considered statistically significant.

### Results

A total number of 166 women were recruited to the study. Respectively, the median age, gravidity and gestational age were 27 years, 2, and 26 weeks. Twenty (12%) of these women had gestational diabetes mellitus, 109 (65.7%) women were *H. pylori* IgG seropositive. Univariate analysis showed that the mean fasting blood sugar (FBS), the median of first hour post prandial blood sugar (1hpp), the median of the 2 h post prandial blood sugar (2hpp) and the proportion of the *H. pylori*-positive patients were significantly higher among those who have gestational diabetes mellitus (P value = 0.010, < 0.001, < 0.001, 0.038). The Log HOMA-B% was lower (0.028) respectively. There was no difference between the gestational diabetes mellitus group and the other group in terms of age, education, employment, gravidity, parity, gestational age, BMI, haemoglobin and QUICKI index results. See Table 1.

**Table 1:** Demographic characteristics of Sudanese pregnant women based on the presence or absence of gestational diabetes mellitus (GDM)

Variables	GDM N = 20	NO GDM N = 146	P value
Age (median) years	26.5	27	0.786
Residence			
Urban	15	108	0.922
Rural	5	38	
Education			
Less than secondary	4	29	0.989
More than secondary	16	117	
Job			
Housewife	15	111	0.920
Employed	5	35	
Gravidity(median)	1	2	0.253
Parity (median)	0	0	0.736
Gestational age (median)/week	26	26	0.552
BMI (median) kg/m^2^	26.24	26.8	0.590
Fasting blood sugar mean (sd) mg/dl	77.3 (16.4)	67.5 (10.5)	0.010
1 h post prandial (median) mg/dl	164.5	129	< 0.001
2 h post prandial (median) mg/dl	163	110	< 0.001
*H. Pylori* positive	9	10	0.038
Haemoglobin (median) g/dl	10.9	10.9	0.873
Log HOMA-B mean (sd)%	1.7 (0.6)	1.99 (0.5)	0.028
Log QUICKI (median)	0.62	0.62	0.403

In multivariate analysis, a higher seropositivity *of H. pylori* was found to be a risk factor for the development of gestational diabetes mellitus (OR = 2.8, 95% CI 1.1 − 7.5, P = 0.036). See Table 2.

**Table 2:** Multivariate logistic regression of the predictors of GDM among pregnant Sudanese women

Variables	OR	CI	P value
Age	1.0	0.9–1.1	0.644
Gravidity	0.9	0.4–2.0	0.778
Parity	1.3	0.5–3.8	0.611
Education	1.2	0.3–3.9	0.822
BMI	1.0	0.9–1.1	0.742
*H. Pylori*	2.8	1.1–7.5	0.036
Haemoglobin	0.8	0.4–1.6	0.517

### Discussion

The main finding in the current study was the association between gestational diabetes mellitus and *H. pylori* IgG seropositivity, where the gestational diabetes group was more likely to be *H. pylori* IgG seropositive. Wang et al. concluded in their meta-analysis that included 39 studies involving 20,000 subjects that there is an association between *H. pylori* infection and diabetes mellitus [26]. Cardaropoli et al. found a strong association between GDM and *H. pylori* among 2820 pregnant women [20]. A large Chinese cohort of more than 30,000 showed an increased association between *H. pylori* infection and type 2 diabetes mellitus among middle-aged people [27]. In their recent publication of 2016, Bonfigli et al. demonstrated that the elimination of *H. pylori* infection improved glucose metabolism in patients with type 2 diabetes [28]. On the other hand, a study from Nigeria concluded that type 2 diabetes was not statistically different between *H. pylori*-infected subjects and controls. However, this study was not powered enough to come out with solid conclusions [29].

In our current study, we found no significant association between the QUICKI index and *H. pylori* IgG seropositivity among the pregnant Sudanese women. Gerig et al. reported a similar finding among 370 severely obese European subjects from both genders [17]. In the same line, a Lebanese study described no association between *H. pylori* and insulin resistance among Lebanese adults. In this study, they used stored blood samples of subjects participating in a national non-communicable disease survey [19]. Likewise, no association between *H. pylori* infection and insulin resistance was documented in a systematic review that included 9 studies with a total number of 2120 subjects [30]. On the other hand, a number of researchers reported the association between *H. pylori* infection and insulin resistance [11, 12, 18, 31].

The association between *H. pylori* infection and insulin resistance can be explained by some biological mechanisms. First, changes in glucose metabolism might lead to chemical alterations in the gastric mucosa that facilitates the detection of *H. pylori* infection [32]. A second explanation is an increase in the proinflammatory cytokines levels in response to *H. pylori* gastric infection that results in structural alterations to the insulin receptors inhibiting their interaction with insulin [33]. He et al. in their study among mice suggested an interaction between *H. pylori* and gut microbiota resulting in an increased insulin resistance [34].

### Conclusion

The current study concludes that an increased seroprevalence of *H. pylori* is a risk factor for the development of gestational diabetes mellitus.

## Limitations

We acknowledge the need for a larger sample size study.

## Abbreviations

QUICKI: quantitative insulin sensitivity check index
HOMA-*B*: homeostatic model assessment-beta
GDM: gestational diabetes mellitus
BMI: body mass index
IADPSG: Diabetes and Pregnancy Study Groups
(FBS): fasting blood sugar
1hpp: first hour post prandial blood sugar
2hpp: two hours post prandial blood sugar
